# Supplementary material for: Enhanced Photocurrent of the Ag Interfaced Topological Insulator Bi2Se3 under UV- and Visible-Light Radiations
Source: Nanomaterials (Basel). 2021 Dec 10;11(12):3353. doi: 10.3390/nano11123353 (PMC8705254; doi:10.3390/nano11123353)
Supplement: Supplementary file 1 [file nanomaterials-11-03353-s001.zip › nanomaterials-1476922-supplementary.pdf]

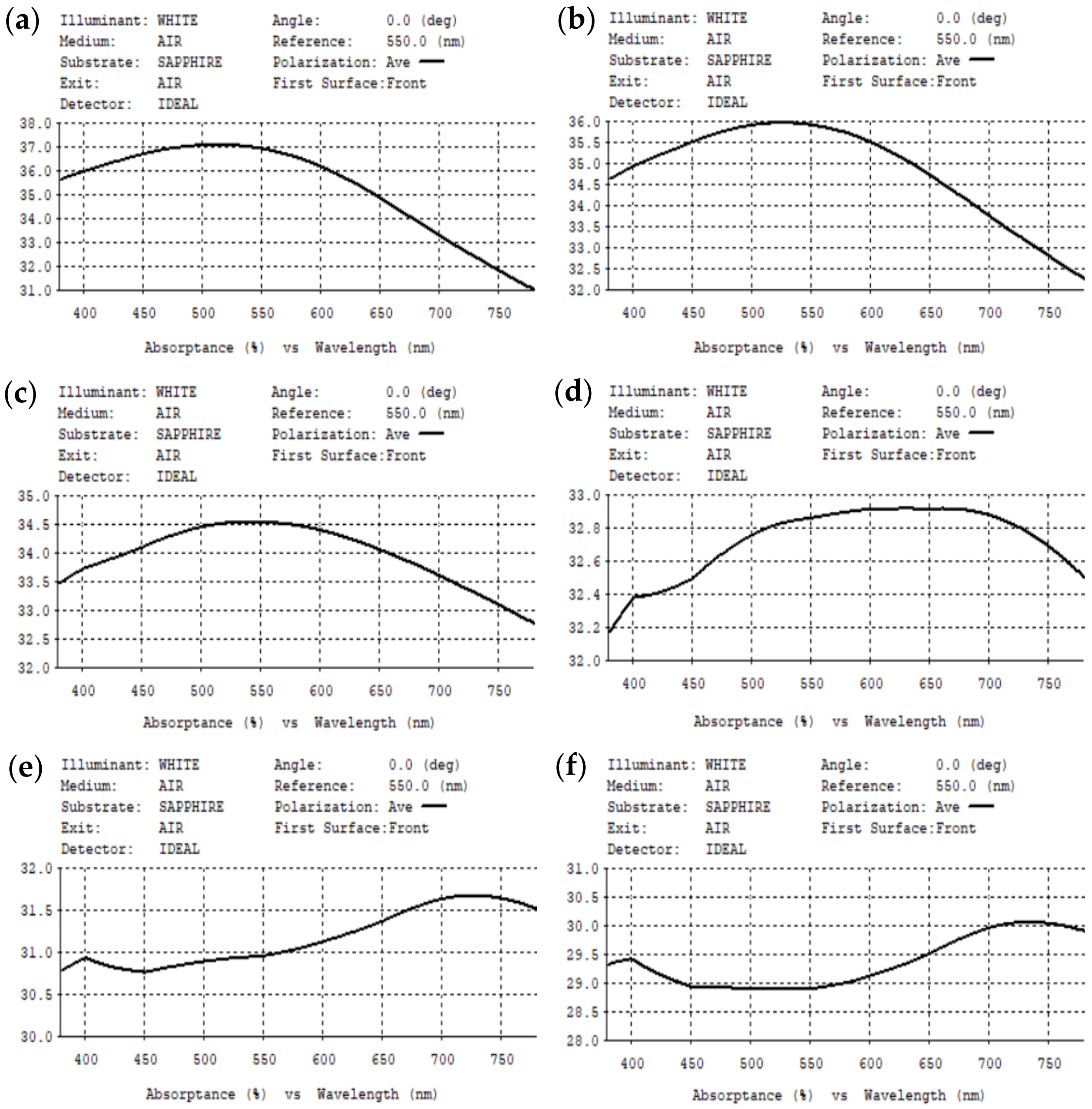

Figure S1. (**a**–**f**) are the simulated absorptance spectra of the Ag@Bi_2Se_3 thin film while Ag thin film at 0, 2, 4, 6, 8, and 10 nm.

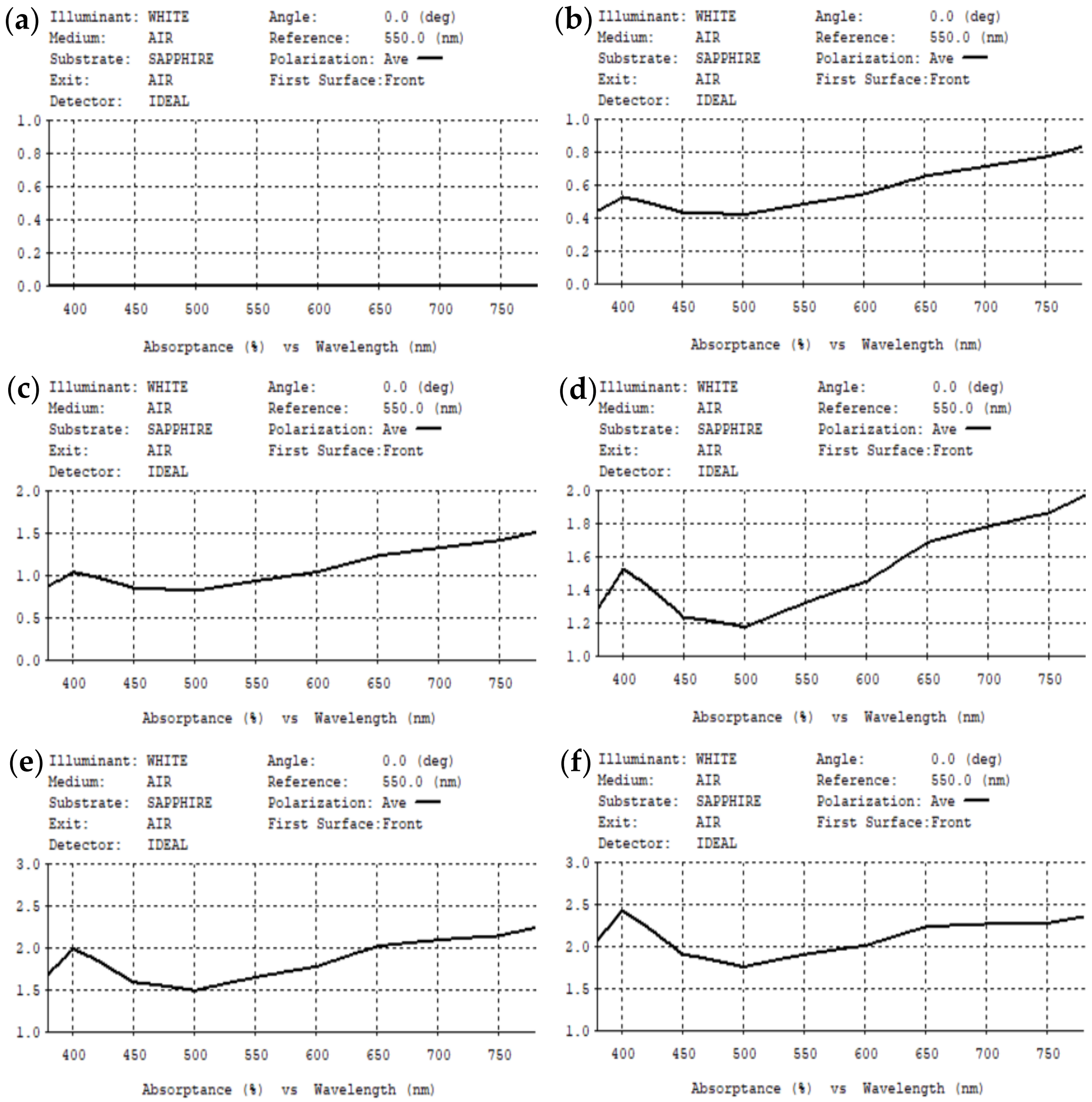

Figure S2. (**a**–**f**) are the simulated absorptance spectra of Ag thin film in 0, 2, 4 , 6, 8, and 10 nm.
